# The Ideal Physician

**Published:** 1892-10

**Authors:** 


### THE IDEAL PHYSICIAN.

---

The following paragraph has been going the rounds of the Journals. Speaking of the ideal physician before a medical class in Baltimore, not long ago, Ex-Secretary of State Bayard said: "I have been thrown in contact with not a few medical men who have been the 'salt of the earth' in their respective communities. It has been my personal fortune to know such a man. It has been my privilege and delight to accompany him in visits where his only medicines were the personal presence and conversation of the man himself. He had shared and had lessened their anxieties; counseled the wayward; cheered the weak-hearted; had rejoiced with them that rejoiced and wept with the weeping. And I have seen such a man so surrounded by an atmosphere of love and trust, holding as it were the heartstrings of a family in his hands, their guide, philosopher and friend; and then I realized what a moral force in society the profession, properly comprehended and properly followed, was capable of exerting, and how relatively small a part of its usefulness was the administration of medicine."

It may be remarked, in this connection, that such a man has not been deemed unworthy of a high place in literature. *Doctor Thorne*, by Anthony Trollope, is perhaps the best description of the ideal physician that has appeared in English. It is a tribute to our profession that the greatest of French writers, Honoré de Balzac, should have made the family physician the hero of his favorite work. *The Country Doctor* is surely a great novel, and in it the ideal practitioner of medicine is beautifully portrayed in the person of kind and noble-hearted old Dr. Benassis. Balzac himself said of this work that it was the gospel in action, and that it was better to have written it than to have made laws or won battles.

